# Examining the Relationship Between Digital Leadership and Leadership Behaviors of Nurse Managers

**DOI:** 10.1155/jonm/1288508

**Published:** 2026-10-17

**Authors:** Miray Celepli Sütbaş, Aysun Ünal

**Affiliations:** ^1^ Kumluca Vocational School Department of Management and Organisation Akdeniz University Antalya Turkey akdeniz.edu.tr; ^2^ Kumluca Faculty of Health Sciences Nursing Management Department Akdeniz University Antalya Turkey akdeniz.edu.tr

**Keywords:** digital leadership, leadership, nursing, technology

## Abstract

**Background:**

The relationship between nurse managers’ leadership behaviors and digital leadership remains insufficiently understood.

**Aims:**

This study aimed to examine the relationship between nurse managers’ employee‐, task‐, and change‐oriented leadership behaviors as perceived by nurses and their perceived overall digital leadership.

**Methods:**

This descriptive cross‐sectional study included 231 nurses working in three public hospitals. Data were collected using the Leadership Behavior Scale and the Digital Leadership Scale. Descriptive and comparative analyses, Pearson correlation analysis, and bivariate simple linear regression were performed.

**Results:**

Task‐oriented leadership received the highest mean score, whereas change‐oriented leadership received the lowest. Overall digital leadership was positively correlated with all leadership behavior dimensions, with the strongest association observed for change‐oriented leadership (*r* = 0.697, *p* < 0.001). In the bivariate regression analysis, change‐oriented leadership was positively associated with overall digital leadership and accounted for 48.6% of its variance (*R* ^2^ = 0.486, *p* < 0.001). Overall digital leadership did not differ significantly according to the demographic and work‐related characteristics examined.

**Conclusions:**

Although task‐oriented leadership received the highest mean score, change‐oriented leadership showed the strongest association with overall digital leadership. These findings highlight the relevance of change‐oriented leadership in digitally transforming healthcare environments; however, the observed association should not be interpreted as causal or as an independent effect over other leadership dimensions.

**Implications for Nursing Management:**

The findings highlight the importance of integrating digital competencies with change‐oriented leadership development in nursing management education and continuing professional development. Supporting nurse managers in developing digital competencies alongside their capacity to guide organizational change may strengthen leadership readiness for increasingly technology‐enabled healthcare environments.

## 1. Introduction

Rapidly evolving technology has become integral to research, development, communication, and other processes across healthcare, contributing to the emergence of digital organizational approaches alongside traditional organizational structures [1]. Technological advances have also brought significant changes to the organization and structure of the nursing profession. From electronic health records and biomedical technologies to artificial intelligence, increasingly complex technologies are transforming modern healthcare and nursing care [2]. In increasingly digital healthcare environments, involving nurses in technology design, maintaining patient‐ and family‐centered care, and addressing nurses’ concerns about workflow are important for ensuring safe and professional practice [3]. There are concerns that if nurses are unable to keep pace with rapid digital transformation, the potential benefits of digital technologies for nursing practice and patient care may not be fully realized [4]. This rapid digital transformation affects not only nursing practice but also the leadership roles of nurse managers and how these roles are enacted [5].

Nurse leaders increasingly use digital technologies to coordinate clinical and organizational processes and maintain communication with nurses, particularly when face‐to‐face interaction is not possible [6]. However, effective leadership in digitally transforming healthcare environments requires more than the use of digital technologies; it also requires leadership processes to adapt to changing work environments.

This transformation of leadership processes was initially conceptualized in the literature through e‐leadership and, more recently, digital leadership. Avolio et al. [7] defined e‐leadership as an information technology–mediated social influence process capable of affecting the attitudes, thoughts, behaviors, and performance of individuals, groups, and organizations. In nursing, e‐leadership has largely been discussed in terms of the combined use of electronic and traditional communication methods [8]. In a qualitative study, Abou Hashish et al. [9] highlighted that effective e‐leadership requires nurse managers not only to possess digital and technical competencies but also to embrace change and actively lead healthcare teams through digital transformation. However, digital transformation extends beyond technology‐mediated leader–follower interactions to encompass organizational strategies, work processes, human resources, and ways of working. This broader transformation has contributed to the development of digital leadership as a concept extending beyond the technology‐mediated interactions emphasized in e‐leadership. Nevertheless, the conceptual boundaries between e‐leadership and digital leadership remain unclear, with some studies using the terms interchangeably and definitions of digital leadership continuing to vary [10, 11].

Recent literature conceptualizes digital leadership as extending beyond technology‐mediated communication. Lin [11] identifies vision‐based motivation, digital empowerment, innovation and entrepreneurship, cross‐boundary collaboration, and dynamic adaptation as key characteristics of digital leadership, while digital strategic thinking, digital insight, digital change, and digital talent development are identified as core dimensions. Consistent with this broader perspective, Tigre et al. [12] suggest that e‐leadership has evolved into digital leadership, reflecting a shift from electronically mediated communication toward effective leadership in digital environments. Taken together, these perspectives suggest that digital leadership is characterized not simply by the use of technology in leadership activities but by the integration of technological, human, strategic, and organizational dimensions of digital transformation within leadership processes.

Understanding the digital leadership capacities of nurse managers in the context of digital transformation also requires consideration of how these capacities relate to broader leadership behaviors. Among employee‐, task‐, and change‐oriented leadership behaviors, change‐oriented leadership particularly intersects conceptually with digital leadership through its emphasis on vision, innovation, adaptation, and organizational change. When Ekvall and Arvonen’s [13] conceptualization of change‐oriented leadership is considered alongside Lin’s [11] synthesis of digital leadership, the two constructs appear to overlap in their emphasis on vision, change and development, creativity and innovation, and adaptation to changing organizational conditions. However, the extent to which digital leadership represents a conceptually distinct leadership construct remains debated. Diniz et al. [14], for example, suggest that digital leadership may also be understood as established leadership attributes adapted to a digital context. Nevertheless, change‐oriented leadership represents a broader orientation toward change and development, whereas digital leadership is specifically situated within the context of digital transformation. Accordingly, this study treats digital leadership and change‐oriented leadership as related but distinct constructs.

A previous study by our research team examined leadership behaviors among nurses working in the same three public hospitals in the region using the Leadership Behavior Scale [15]. Although the studies were conducted in the same institutional settings, participant‐level identifiers were not retained in a manner that permits individual matching across the two datasets; therefore, the extent of participant overlap cannot be determined. Importantly, the present study addresses a distinct research question by examining overall digital leadership and its relationship with employee‐, task‐, and change‐oriented leadership behaviors in the context of healthcare digital transformation. Based on this conceptual framework, this study aimed to examine the relationship between nurse managers’ employee‐, task‐, and change‐oriented leadership behaviors as perceived by nurses and their perceived overall digital leadership. The study addressed the following research questions:

1. What are nurses’ perceptions of their managers’ leadership behaviors and overall digital leadership?
2. Do these perceptions differ according to nurses’ sociodemographic and work‐related characteristics?
3. Are nurses’ perceptions of their managers’ leadership behaviors associated with their perceptions of their managers’ overall digital leadership?

## 2. Materials and Methods

### 2.1. Study Design

This descriptive cross‐sectional study was conducted between May 2023 and August 2024 at three state hospitals located in southern Türkiye.

### 2.2. Study Participants

The study population consisted of 346 nurses employed at the three hospitals. Participation was voluntary, and convenience sampling was used. A total of 231 nurses participated in the study. Participants reported their work‐unit category; however, identifiers for individual nurse managers or specific clinical units were not collected. Therefore, the number of distinct nurse managers rated could not be determined, and observations could not be clustered at the individual manager or specific‐unit level. An a priori sample‐size calculation was based on the primary correlational analysis. Assuming a two‐tailed significance level of 0.05, 80% statistical power, and a medium effect size (*r* = 0.30), a minimum sample size of 84 participants was required. The final sample of 231 nurses exceeded this requirement.

### 2.3. Instruments

#### 2.3.1. Sociodemographic Information Form

The sociodemographic and work characteristics form developed by the researchers consisted of eight questions addressing age, gender, marital status, educational level, years of professional experience, work unit, work pattern, and weekly working hours.

#### 2.3.2. The Digital Leadership Scale

The Digital Leadership Scale was developed by Zeike et al. [16] and adapted into Turkish by Sürücü, Yıkılmaz, and Maşlakçı [17]. The scale consists of six items rated on a 5‐point Likert scale, with higher scores indicating higher perceived levels of digital leadership. The Turkish adaptation supported a one‐factor structure and reported high internal consistency (Cronbach’s *α* = 0.90). In the present sample, the total Digital Leadership Scale also demonstrated excellent internal consistency (Cronbach’s *α* = 0.927). Confirmatory factor analysis was conducted to examine the one‐factor structure in the present sample. Standardized factor loadings ranged from 0.698 to 0.894 (all *p* < 0.001). The model yielded *χ* ^2^(9) = 68.40, *p* < 0.001, CFI = 0.945, TLI = 0.909, and RMSEA = 0.169, 90% CI [0.133, 0.208]. Given the elevated RMSEA, the model fit was not uniformly satisfactory. Nevertheless, considering the published one‐factor Turkish adaptation, the substantial factor loadings, and the absence of clear empirical support for treating the items as separate dimensions, the overall Digital Leadership Scale score was used in subsequent analyses.

#### 2.3.3. Leadership Behavior Scale

The Leadership Behavior Scale, developed by Ekvall and Arvonen [18], consists of 36 items assessing three dimensions of leadership behavior: employee‐oriented leadership (14 items), task‐oriented leadership (12 items), and change‐oriented leadership (10 items). The scale was adapted into Turkish by Tengilimoğlu [19], who reported a Cronbach’s alpha coefficient of 0.96. In the present sample, the total Leadership Behavior Scale demonstrated excellent internal consistency (Cronbach’s *α* = 0.987). The employee‐oriented, task‐oriented, and change‐oriented leadership subscales also demonstrated high internal consistency, with Cronbach’s *α* values of 0.965, 0.963, and 0.950, respectively.

The Leadership Behavior Scale is scored using a 5‐point Likert‐type scale: (1) Strongly disagree, (2) Disagree, (3) Undecided, (4) Agree, and (5) Strongly agree. There are no negative items in the scale. The mean score method is used for scale evaluation.

### 2.4. Ethical Considerations

Ethical approval was obtained from the Akdeniz University Clinical Research Ethics Committee (Approval No. 596221/106‐6; March 6, 2023). The study was conducted in accordance with the ethical principles of the Declaration of Helsinki. At the beginning of the online survey, participants were informed that participation was voluntary. Only those who provided electronic informed consent were able to proceed to the survey questions.

### 2.5. Statistical Analysis

Descriptive statistics were used to summarize participant characteristics and study variables. Independent‐samples *t*‐tests and one‐way analyses of variance were used to examine differences in leadership and digital leadership scores according to sociodemographic and work‐related characteristics. Because of unequal group sizes and evidence of heterogeneity of variance for some comparisons involving length of service, Welch’s ANOVA was used, followed by Games–Howell post hoc tests for pairwise comparisons. Eta‐squared (*η* ^2^) values were reported as effect‐size estimates. Pearson correlation analysis was used to examine associations between leadership behaviors and overall digital leadership.

A bivariate simple linear regression analysis was conducted to further examine the association between change‐oriented leadership and overall digital leadership. Change‐oriented leadership was entered as the sole predictor and overall digital leadership as the outcome. Preliminary models simultaneously including employee‐, task‐, and change‐oriented leadership indicated substantial multicollinearity; therefore, the leadership dimensions were not entered concurrently. The regression analysis was interpreted as a bivariate association and not as evidence of an independent or causal effect of change‐oriented leadership. Model fit was evaluated using *R* ^2^, adjusted *R* ^2^, and the F statistic, and regression coefficients were reported as unstandardized coefficients (B), standard errors (SE), standardized coefficients (β), and 95% confidence intervals. Statistical significance was set at *p* < 0.05.

## 3. Results

Among the participating nurses, 86.0% were female, 53.7% were married, and 76.6% held a bachelor’s degree. Regarding professional experience, 34.6% had worked for 1–5 years and 23.8% for less than 1 year. One‐third of the participants (33.3%) worked in intensive care units or emergency departments, 22.5% worked in internal medicine units, 61.0% worked rotating day/night shifts, and 54.5% worked 40–60 h per week. The mean age of the participants was 31.94 years (Table 1).

**TABLE 1:** Descriptive and occupational characteristics of nurses (*N* = 231).

Characteristics	*N*	%
Gender		
Female	199	86.1
Male	32	13.9
Marital status		
Single	107	46.3
Married	124	53.7
Education status		
High school	11	4.8
License	177	76.6
Master’s degree	39	16.9
PhD	4	1.7
Length of service in the profession		
0‐1 years	55	23.8
1–5 years	80	34.6
5–10 years	48	20.8
10–15 years	17	7.4
15–20 years	16	6.9
Over 20 years	15	6.5
Unit		
Internal service	52	22.5
Surgical service	39	16.9
Intensive care/emergency	77	33.3
Management	32	13.9
Polyclinic units	31	13.4
Mode of operation		
Day	82	35.5
Day/night	141	61.0
Night	8	3.5
Working hours		
40 h	77	33.3
40–60 h	126	54.5
60 and above	28	12.1
Average age	31.944	
Total		100

Among the leadership behavior dimensions, task‐oriented leadership had the highest mean score (3.19 ± 0.98), whereas change‐oriented leadership had the lowest (2.94 ± 0.98). The mean overall digital leadership score was 2.68 ± 0.87 (Table 2).

**TABLE 2:** Mean scores of leadership behaviors and digital leadership.

Leadership and its subdimensions	M ± SD	Digital leadership	M ± SD
Task oriented	**3.19 ± 0.98**	Digital Leadership Scale	2.68 ± 0.87
Change oriented	**2.94 ± 0.98**		
Employee oriented	2.98 ± 1.10		
Leadership Behavior Scale	3.04 ± 1.00		

*Note:* The bold values indicate the highest and lowest mean scores among the leadership behavior subdimensions. Specifically, task‐oriented leadership had the highest mean score (3.19 ± 0.98), whereas change‐oriented leadership had the lowest mean score (2.94 ± 0.98). M = mean.

Abbreviation: SD, standard deviation.

Leadership behavior scores differed significantly according to length of service for overall leadership (Welch’s F(5, 60.6) = 5.21, *p* < 0.001, *η* ^2^ = 0.085), task‐oriented leadership (Welch’s F(5, 60.7) = 5.86, *p* < 0.001, *η* ^2^ = 0.092), employee‐oriented leadership (Welch’s F(5, 60.3) = 5.08, *p* < 0.001, *η* ^2^ = 0.083), and change‐oriented leadership (Welch’s F(5, 60.1) = 4.05, *p* = 0.003, *η* ^2^ = 0.070). Games–Howell post hoc comparisons indicated that the significant pairwise differences primarily involved the 5–10‐year experience group, which generally reported lower leadership scores than several other experience groups. Given the small sample sizes in some experience categories, these subgroup findings should be interpreted cautiously (Table 3).

**TABLE 3:** Comparison of the relationship between some descriptive and working characteristics of nurses and their leadership behaviors subscales (*N* = 231).

Characteristics	LBS mean ± SD	F/*t*	*p*	Task oriented mean ± SD	*F*/*t*	*p*	Employee oriented mean ± SD	*F*/*t*	*p*	Change oriented mean ± SD	F/*t*	*p*
Gender												
Female	3.04 ± 0.99	*t* = 0.199		3.19 ± 0.97	*t* = 0.056		2.99 ± 1.09	*t* = 0.228	0.820	2.94 ± 0.98	0.306	0.760
Male	3.01 ± 0.97		0.842	3.18 ± 1.07		0.955	2.94 ± 1.10			2.89 ± 1.02		
Education status												
High school	3.21 ± 1.02	*F* = 0.934	0.425	3.31 ± 1.30	*F* = 0.567	*p* = 0.637	3.00 ± 1.29	1.62	0.366	3.30 ± 1.04	1.319	0.269
License	3.06 ± 0.96			3.20 ± 0.94			3.00 ± 1.05			2.95 ± 0.94		
Master’s degree	2.99 ± 1.12			3.18 ± 1.11			2.94 ± 1.19			2.83 ± 1.12		
PhD	2.27 ± 0.70			2.58 ± 0.64			2.03 ± 0.81			2.25 ± 0.70		
Length of service in the profession												
0‐1 year	3.34 ± 1.0	Welch F(5, 60.6) = 5.21	*p* < 0.001	3.52 ± 0.90	Welch F(5, 60.7) = 5.86,	*p* < 0.001	3.28 ± 1.18	Welch F(5, 60.3) = 5.08,	*p* < 0.001	3.21 ± 0.95	Welch F(5, 60.1) = 4.05	*p* = 0.003
1–5 years	2.90 ± 0.99			3.08 ± 1.02			2.86 ± 1.05			2.80 ± 0.99		
5–10 years	2.68 ± 0.96			2.84 ± 0.94			2.61 ± 1.03			2.60 ± 0.98		
10–15 years	2.81 ± 1.1			2.89 ± 1.14			2.66 ± 1.27			2.91 ± 1.10		
15–20 years	3.50 ± 0.78			3.55 ± 0.79			3.58 ± 0.79			3.31 ± 0.87		
Over 20 years	3.49 ± 0.56			3.70 ± 0.53			3.41 ± 0.68			3.34 ± 0.57		
Unit	3.01 ± 0.88			3.13 ± 0.87			2.97 ± 0.99			2.93 ± 0.87		
Internal service	3.12 ± 0.95	*F* = 0.488	0.746	3.28 ± 0.94	*F* = 0.548	0.679	3.09 ± 1.06	0.463	0.752	2.96 ± 0.90	0.457	0.761
Surgical service	2.95 ± 1.11			3.101 ± 1.07			2.88 ± 1.20			2.85 ± 1.11		
ICU/emergency	3.03 ± 1.04			3.22 ± 1.02			2.92 ± 1.15			2.93 ± 0.99		
Management polyclinic units	3.22 ± 0.94			3.37 ± 0.95			3.16 ± 1.04			3.13 ± 0.94		

*Note:* Values are presented as mean ± standard deviation. *t* = independent‐samples t‐test; *F* = one‐way analysis of variance (ANOVA) or Welch’s ANOVA, as specified in the table. Statistical significance was set at *p* < 0.05.

Abbreviations: LBS, Leadership Behavior Scale; SD, standard deviation.

No statistically significant differences in overall digital leadership scores were observed according to gender, educational level, length of service, or work unit (all *p* > 0.05) (Table 4).

**TABLE 4:** Comparison of overall digital leadership scores according to nurses’ demographic and work‐related characteristics (*N* = 231).

Characteristics	DLmean ± SD	F/*t*	*p*
*Gender*			
Female	2.66 ± 0.85	*t* = −0.309	0.757
Male	2.71 ± 0.1.01		

*Education status*			
High school	2.74 ± 0.90	*F* = 2.747	0.066
License	2.73 ± 0.85		
Master’s degree	2.38 ± 0.93		

*Length of service in the profession*			
0‐1 year	2.89 ± 0.76	*F* = 2213	0.054
1–5 years	2.56 ± 0.88		
5–10 years	2.50 ± 0.98		
10–15 years	2.59 ± 1.11		
15–20 years	2.58 ± 0.65		
Over 20 years	3.12 ± 0.56		

*Unit*			
Internal service	2.72 ± 0.79	*F* = 2.235	0.066
Surgical service	2.66 ± 0.94		
ICU/emergency	2.46 ± 0.82		
Management	2.79 ± 1.0		
Polyclinic units	2.96 ± 0.84		

*Note:* M = Mean. *a* = *t* paired sample test *b* = *t* independent sample test, *F* = one way ANOVA test, *p* < 0.05, *p* < 0.01.

Abbreviation: SD, standard deviation.

Pearson correlation analysis showed positive and statistically significant associations between overall digital leadership and all leadership behavior dimensions. Overall digital leadership was correlated with task‐oriented leadership (*r* = 0.629, *p* < 0.001), employee‐oriented leadership (*r* = 0.614, *p* < 0.001), and change‐oriented leadership (*r* = 0.697, *p* < 0.001). The strongest association was observed with change‐oriented leadership (Table 5).

**TABLE 5:** Correlations between leadership behaviors and overall digital leadership (*N* = 231).

Leadership behaviors and subdimensions		Task oriented	Employee oriented	Change oriented	LD overall average score
DL overall average score	*r*	0.629^∗∗^	0.614^∗∗^	0.697^∗∗^	0.659^∗∗^
	*p*	< 0.001	< 0.001	< 0.001	< 0.001

*Note: r* = correlation, *p* < 0.05.

^∗∗^ *p* < 0.01.

A bivariate simple linear regression analysis showed that change‐oriented leadership was positively associated with overall digital leadership (*B* = 0.622, SE = 0.042, *β* = 0.697, 95% CI [0.538, 0.705], *p* < 0.001). The model was statistically significant, *F* (1, 229) = 216.00, *p* < 0.001, and accounted for 48.6% of the variance in overall digital leadership (*R* ^2^ = 0.486, adjusted *R* ^2^ = 0.483) (Table 6). Because change‐oriented leadership was entered as the sole predictor, this analysis represents a bivariate association and does not demonstrate an independent effect over the other highly correlated leadership dimensions. Regression diagnostics indicated no highly influential observations (maximum Cook’s distance = 0.116). The residuals did not significantly deviate from normality (Shapiro–Wilk *W* = 0.988, *p* = 0.061), and visual inspection of the Q–Q and residual plots indicated no substantial violations of the regression assumptions.

**TABLE 6:** Bivariate simple linear regression analysis of change‐oriented leadership and overall digital leadership.

Outcome	B	SE	β	95% CI for B	*R* ^2^	Adjusted *R* ^2^	F	*p*
Overall digital leadership	0.622	0.042	0.697	[0.538, 0.705]	0.486	0.483	216.00	< 0.001

*Note: N* = 231. Change‐oriented leadership was entered as the sole predictor. The model represents a bivariate association and should not be interpreted as demonstrating an independent effect of change‐oriented leadership after controlling for the other leadership dimensions. *B* = unstandardized regression coefficient; *β* = standardized regression coefficient. *F* (1, 229) = 216.00.

Abbreviations: CI, confidence interval; SE, standard error.

## 4. Discussion

This study examined the relationship between leadership behaviors perceived by nurses in their managers and their perceptions of their managers’ overall digital leadership. Task‐oriented leadership received the highest mean score, whereas change‐oriented leadership received the lowest. The mean overall digital leadership score was 2.68 ± 0.87. This pattern may reflect the structured and hierarchical workflows prevalent in healthcare settings, where operational continuity and process management are strongly emphasized. Nevertheless, rapidly changing healthcare environments also require leaders to develop a digital vision, adapt to change, and involve employees in organizational transformation [16, 20, 21]. Previous nursing research has similarly highlighted the growing importance of digital leadership in technology‐enabled healthcare environments [22]. Healthcare leaders have been described as supporters, change managers, advocates, project managers, facilitators, and champions in health information technology implementation, while also requiring adequate knowledge and support to fulfill these roles effectively [23]. Taken together, these findings reinforce the importance of developing digital competence alongside change‐management, innovation, and leadership capabilities.

Leadership behavior scores differed according to nurses’ length of professional experience, with significant differences observed across overall, task‐oriented, employee‐oriented, and change‐oriented leadership. Post hoc comparisons indicated that these differences primarily involved the 5–10‐year experience group, which generally reported lower leadership scores than several other experience groups. However, the pattern was not linear across increasing levels of professional experience, and the relatively small numbers of participants in some longer‐service categories warrant cautious interpretation. In contrast, overall digital leadership did not differ significantly according to gender, educational level, length of service, or work unit. These findings suggest that perceptions of managers’ digital leadership were relatively consistent across the demographic and work‐related groups examined in this sample.

Overall digital leadership was positively associated with all three leadership behavior dimensions, with the strongest correlation observed for change‐oriented leadership (*r* = 0.697). This finding is conceptually consistent with the shared emphasis of change‐oriented and digital leadership on vision, innovation, adaptation, and organizational change [11, 13]. This interpretation is also consistent with Abou Hashish et al. [9], who emphasized that effective e‐leadership among nurse managers involves not only digital and technical competencies but also embracing change and guiding healthcare teams through digital transformation. The bivariate regression analysis further showed that change‐oriented leadership accounted for 48.6% of the variance in overall digital leadership. However, because change‐oriented leadership was examined as the sole predictor and the leadership dimensions were highly intercorrelated, this finding should not be interpreted as demonstrating an independent effect of change‐oriented leadership over employee‐ or task‐oriented leadership. Rather, nurses who perceived their managers as more change oriented also tended to perceive them as stronger digital leaders. This pattern supports the conceptual relevance of change‐oriented leadership in digitally transforming healthcare environments while maintaining the distinction between the two constructs.

These findings should be interpreted cautiously because leadership behaviors and overall digital leadership were assessed from the same respondents at the same time, potentially increasing shared‐method effects. Moreover, the cross‐sectional design does not permit causal inference. These considerations are addressed further in the limitations of the study.

## 5. Limitations

This study has several limitations. First, it was conducted among nurses from three public hospitals in a single region of Türkiye, which may limit the generalizability of the findings. Individual nurse–manager and specific clinical‐unit identifiers were not collected; therefore, potential clustering within managers or units could not be accounted for statistically. Second, leadership behaviors and digital leadership were assessed through self‐reports from the same respondents at the same time, introducing potential response bias and common method variance. Third, organizational factors such as culture, technological infrastructure, and digital maturity were not measured, and some subgroup analyses involved small sample sizes and should, therefore, be interpreted cautiously. Finally, the cross‐sectional design precludes causal or directional inference, and the bivariate regression should not be interpreted as demonstrating an independent effect of change‐oriented leadership over the other highly correlated leadership dimensions. Future longitudinal, multicenter, and multisource studies that account for organizational and managerial clustering are warranted.

## 6. Conclusions

This study demonstrated positive associations between nurses’ perceptions of their managers’ leadership behaviors and overall digital leadership. Although task‐oriented leadership received the highest mean score, change‐oriented leadership showed the strongest association with overall digital leadership. The bivariate regression analysis further demonstrated a substantial association between change‐oriented leadership and overall digital leadership; however, this finding should not be interpreted as causal or as evidence of an independent effect over the other leadership dimensions.

These findings suggest that change‐oriented leadership may be particularly relevant in digitally transforming healthcare environments. Developing nurse managers’ digital competencies alongside their capacity to support innovation, adaptation, and organizational change may, therefore, be important for leadership development. Future longitudinal, multicenter, and multisource studies should examine how leadership behaviors, organizational context, and digital maturity interact during healthcare digital transformation.

## 7. Implications for Nursing Management

The findings highlight the importance of integrating digital competencies with broader leadership development in nursing management. In particular, the strong association between change‐oriented leadership and overall digital leadership suggests that leadership development initiatives may benefit from combining digital competency development with skills related to innovation, adaptation, and organizational change. Healthcare organizations should provide nurse managers with continuing professional development, mentoring, and opportunities to participate in digital transformation initiatives. Such support may strengthen their readiness to guide nursing teams through increasingly technology‐enabled healthcare environments.

## Author Contributions

Aysun Ünal and Miray Celepli Sütbaş: conceptualization, methodology, formal analysis, and writing–original draft. Aysun Ünal: investigation, data curation, validation, supervision, and methodology (critical revisions). Miray Celepli Sütbaş: writing–review and editing and final approval.

## Funding

No funding was received for this manuscript.

## Conflicts of Interest

The authors declare no conflicts of interest.

## Data Availability

The data supporting the findings of this study are available from the corresponding author upon reasonable request.
